# Trace Elements in Soils and Selected Agricultural Plants in the Tongling Mining Area of China

**DOI:** 10.3390/ijerph15020202

**Published:** 2018-01-25

**Authors:** Ziwei Ding, Yang Li, Qingye Sun, Haojie Zhang

**Affiliations:** School of Resources and Environmental Engineering, Anhui University, Hefei 230601, China; dingziwei819@163.com (Z.D.); ly17019@ahu.edu.cn (Y.L.); blossom_shadow@sina.com (H.Z.)

**Keywords:** trace elements, vegetable soils, fruit vegetables, health risk, mining area

## Abstract

The quality and safety of agricultural products from metal mining areas are of wide concern. In order to investigate the contents and health risks of trace elements in fruit vegetables planted in metal mining areas, 440 samples of fruit vegetables from 117 vegetable plots were collected from Tongling mining area. Trace element contents in fruit vegetables and soil were measured. The results indicated that the total concentration of trace elements in some of the soil samples exceeded the Grade II national standard in China. Transfer factor (TF) of Cd was the highest (8.360), followed by Zn, Cu, As, and Pb. Estimated daily intake (EDI) of the trace elements, except Cd, were generally below the maximum tolerable daily intake (MTDI). The target hazard quotient (THQ) of Zn for children was more than 1 in some vegetables, suggesting potential health hazards for child population. Total target hazard quotient (TTHQ) of Cu and Zn were also more than 1 through consumption of all vegetables, indicating significant health risks. For both adults and children, hazard index (HI) was more than 1 for the consumption of vegetables. The findings reveal the health risks associated with the consumption of trace elements through the intake of selected vegetables in the population of Tongling.

## 1. Introduction

Human health is closely related to the soil quality and especially to the degree of soil pollution [1,2], thus soil contamination has attracted a great deal of attention in the world. Soil acts as a sink and also as a source of pollution with the capacity to transfer pollutants to groundwater and the food chain, and then to humans and/or animals [3]. Many crops can accumulate trace elements, thereby potentially resulting in the rise in the metal contents of agricultural products [4,5,6,7,8,9]. Vegetables, as an important part of people’s diets, contain a range of both essential and toxic elements [10,11,12,13]. Trace elements, such as Cu, Zn, Pb, Cd, and As might be toxic and accumulate in the human body over a long time. Contaminated vegetables can cause serious clinical and physiological problems for humans, especially when consumed in large quantities [14]. The ingestion of Cd contaminated food can cause both chronic and acute health effects, such as bone fractures, kidney dysfunction, hypertension, and cancer [15]. Zn can cause a sideroblastic anemia in spite of the need for Zn, which contributes to important for body functions [16]. Pb is a common trace element, and excessive intake is associated with cardiovascular, nervous, and bone diseases [17]. High contents of Cu, Cd, and Pb in vegetables are related to high prevalence of upper gastrointestinal cancer [18].

Tongling City, a mining city of non-ferrous metal ores in China, has a very long mining history, and there are many active and abandoned mine wastelands in this area [19]. Elevated levels of trace elements have been reported in the surrounding environment of mining cities [20,21]. Although the relative contribution of trace elements has not yet been clearly established, the dietary intake is considered to be the critical exposure pathway [14]. Many studies have confirmed that mining and processing of non-ferrous metal ores can result in the trace element pollution of soils and waterbodies. The trace elements from mine wastelands can enter into the farmlands located close to mine wastelands by surface runoff, wind blow, and irrigation, which may pose a potential risk to human health [22]. A previous study by Xu et al. found that some leaf vegetables planted in mining areas have higher associated health risks [12]. Fruit vegetables are an important type of vegetable in people’s diets. However, there have not been detailed studies assessing the potential health risks of consuming fruit vegetables from mining areas.

In this study, eight fruit vegetables belonging to families Solanaceae, Leguminosae, and Cucurbitaceae were sampled from a mining area and their trace elements were determined. The main aim of this study was to: (1) calculate the estimated daily intake (EDI), target hazard quotient (THQ), and hazard index (HI) of trace elements via consumption of these vegetables; (2) evaluate the potential health risks to local consumers in the study area; and suggest fruit vegetables with lower health risks.

## 2. Materials and Methods

### 2.1. Description of the Study Area

**Figure 1:**
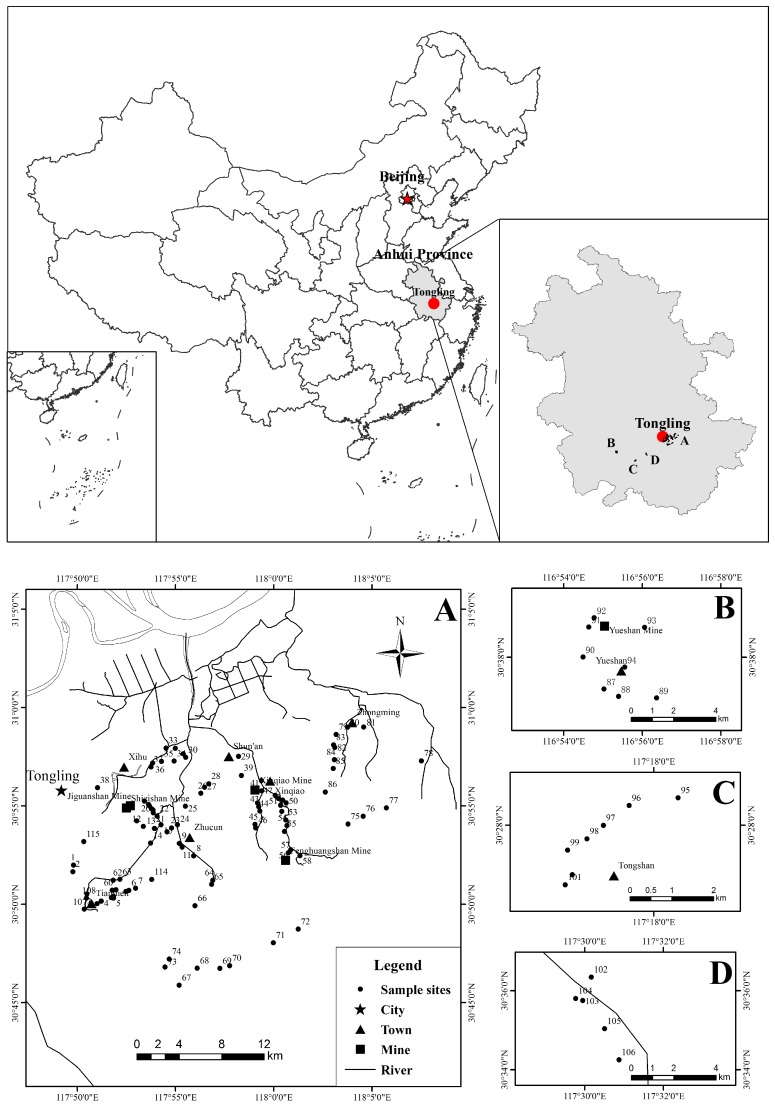
Location map of sample collection in the study area. (A) Concentrated areas of mining; (B) Yueshan Copper Mine; (C) Tongshan Copper Mine; (D) Unknown Copper Mine.

Tongling, located in the southern bank of the Yangtze River, has a subtropical monsoon climate, with an annual average temperature of 16.2 °C and the summer average temperature of 27.4 °C. On average, the frost-free period is 237–258 days, and rainfall is abundant, with the mean annual precipitation of 1390 mm and mean annual humidity of 75–81%. The farmlands are distributed mainly in the northern areas adjoining to the Yangtze River and the southern valley along the rivers in Tongling City (Figure 1). The main crops include rice, wheat, and rape and the pattern of crop-rotation mainly is rice-wheat rotation or rice-rape rotation. Additionally, there are many plots where residents plant vegetables. The vegetables planted in these plots are diverse, mainly belonging to the following families: Cruciferae, Leguminosae, Solanaceae, Compositae, Cucurbitaceae, and Liliaceae.

### 2.2. Sample Collection and Pre-Treatment

In this study, a total of 117 soil samples (depth 0–20 cm) were collected with a polyvinylchloride (PVC) pipe; at each sampling site, five sub-samples were collected and thoroughly mixed to form a soil sample. The sample was sealed in a polyethylene bag and brought to the laboratory and pre-treated as reported by Xu et al. [12]. Samples of fruit vegetables were collected according to actual situation from the same location where the soil samples were collected. The total number of vegetables was 440, including *Capsicum annuum* (Solanaceae), *Solanum melongena* (Solanaceae), *Lycopersicon esculentum* (Solanaceae), *Vigna sesquipedalis* (Leguminosae), *Glycine max* (Leguminosae), *Dolichos lablab* (Leguminosae), *Luffa cylindrica* (Cucurbitaceae), and *Cucumis sativus* (Cucurbitaceae).

### 2.3. Chemical Analysis

Soil pH was measured in water (1 g:2.5 mL soil:water) using an electronic pH meter (PB-10, Sartorius, Goettingen, Germany). Soil organic matter content was determined by the K_2_Cr_2_O_7_-H_2_SO_4_ oxidation method [23]. For the estimation of total content of trace elements in the soils, 1.00 g soil was combined with 2.35 mL HNO_3_ 65% and 7 mL HCl 37% and digested in a Microwave Digestion System in closed Teflon vessels at 190 °C for 25 min. After the vessels were cooled, the solutions were filtered and diluted to 50 mL with deionized water [24]. Total contents of Cu, Zn, Pb, Cd, and As in the soil samples were determined by Inductively Coupled Plasma-Atomic Emission Spectrometry (ICP-AES, XSP Intrepid II, Thermo, Waltham, MA, USA). The contents of trace elements in vegetable samples were determined by High-Resolution Inductively Coupled Plasma-Mass Spectrometry (HR-ICP-MS, X II, Thermo, Waltham, MA, USA). In the analysis of trace elements, standard reference materials, including soil (GBW07429), cabbage (GSB5), spinach (GSB6), long bean (GSB12), and green tea (GSB30), were used for validation of the analytical procedure. The recovery rates were around 88–110 percent for all of the trace elements in the soil and plant reference materials.

### 2.4. Data Analysis

#### 2.4.1. Transfer Factor from Soil to Edible Parts of Vegetables

Total concentration of trace elements in soils and plants were calculated on the basis of dry weight. The soil-to-plant transfer factor (*TF*) was calculated as *TF* = *C_plants_*/*C_soil_*, where *C_plants_* and *C_soil_* represent the trace element concentration in the edible parts of vegetables and soils, respectively [12].

#### 2.4.2. Estimated Daily Intake (EDI) of Trace Elements

The estimated daily intakes of trace elements through vegetables were calculated by the following formula,

(1) $$EDI=\frac{\sum FIR_{i}\times C_{i}}{BW}$$

where *FIR* is the daily vegetable consumption rate, 154.95 g day^−1^ for adults and 105.5 g day^−1^ for children, *C* is the trace element concentrations (mg/kg, fresh weight) in vegetable, and *BW* is the body weight of individuals, considered to be 60 kg for adults and 16 kg for children [25].

#### 2.4.3. Target Hazard Quotient (THQ)

The target hazard quotient was calculated as

(2) $$THQ=\frac{EFr\times ED\times FIR\times C}{RfD\times BW\times AT}\times 10^{-3}$$

and total target hazard quotient (*TTHQ*) was calculated as

(3) $$TTHQ(individual\;vegetable)=THQ_{element1}+THQ_{element2}+\cdots \cdots +THQ_{elementn}$$

where *EFr* is the exposure frequency (365 days/year); *ED* is the exposure duration (70 years); *FIR* is the rate of ingestion (g/person/day); *C* is the trace element concentration in vegetables (mg/kg, fresh weight); *RfD* is the oral reference dose (mg/kg/day); *BW* is the average body weight; and *AT* is the averaging time for non-carcinogens (365 days/year × number of exposure years, assuming 70 years) [17]. The values of *RfD* for Pb was 0.004, which has been obtained from USEPA 2000 [26]. The values of *RfD* for Cd, As, Zn and Cu were 0.001, 0.0003, 0.3 and 0.04 mg kg^−^^1^ day^−^^1^, respectively, as determined by the China National Environmental Protection Agency (HJ 25.3-2014).

In order to assess the overall potential for non-carcinogenic effects, a hazard index (*HI*) was formulated [17]

(3) $$HI=\sum TTHQ=TTHQ_{food1}+TTHQ_{food2}+\cdots \cdots +TTHQ_{foodn}$$

#### 2.4.4. Statistical Analysis

The mean value, median, range, and coefficient of variation (CV%) were calculated in Microsoft Excel. The relationships and sources of all trace elements were performed by Pearson correlation and cluster analysis by using the statistical package SPSS 19.0 (IBM, Armonk, NY, USA). The level of significance was set at *p* ≤ 0.05 (two-tailed).

## 3. Results and Discussion

### 3.1. Content of Trace Elements in Soils

Table 1 shows the characteristics and concentrations of Cu, Zn, Pb, Cd, and As in the 117 soil samples. In this study, the pH value of most soils ranged between 6.0 and 8.0, with several samples displaying a pH of less than 5.5 or more than 8.5. The pH values play an important role in the speciation and bioavailability of trace elements in soil, and the maximum allowable contents in soil vary with soil pH [27]. The transfer of trace element ions from the soil into vegetables is decreased when the pH > 6.5 [6]. The contents of soil organic matter ranged from 0.7% to 10.5%, and the mean value was 2.7%, which was slightly higher than the results reported by Xu et al. [12] (mean value: 2.5%) in the vicinity of Tongling mining area. Table 1 also shows that there was a significant difference in the contents of trace elements among different soil samples. The CV (coefficient of variation) of five trace elements displayed the following order: As > Cu > Pb > Zn > Cd. According to the Environmental Quality Standard for Soils (Grade II) regulated by State Environment Protection Administration of China, 37.6%, 19.7%, 4.3%, 23.1%, and 24.8% of the soil samples in this study exceeded the maximum allowable level for Cu, Zn, Pb, Cd, and As, respectively. Data analysis showed that there were significantly positive correlations among the five trace elements (Table 2), indicating that the five trace elements in soils have the same source [28]. Further analysis found that the soil samples from the area of mining activities, such as sites 13, 17, 20, 23, 24, 32, 34, 37, 45, 49, 58, etc., have higher contents of the five trace elements, which indicated that the trace elements in soil originated mainly from mining activities.

### 3.2. The Contents of Trace Elements in Vegetables

The fruit vegetable samples in this study belong to eight species from three families grown in the Tongling mining area. The contents of trace elements varied greatly among species and sampling locations. On average, the highest contents of Cu, Zn, and As were found in *G. max*, while none was detected beyond the food safety limit. In the eight fruit vegetables considered in this study, *D. lablab* and *S. melongena* had the highest mean content of Pb and Cd. The Pb contents in *D. lablab* and *L. esculentum* exceeded the food safety limit, at 17% and 10%, respectively. The Cd contents in *C. annuum*, *S. melongena*, and *L. esculentum* belonging to Solanaceae also exceeded the food safety limit, at 5%, 14%, and 3%, respectively. In the same area, Xu et al. [12] found that *Vicia faba* (Leguminosae) accumulated the highest content of Cu and Zn, indicating vegetables of Leguminosae can collect more Cu and Zn in edible parts. However, the Pb average contents in *V. sesquipedalis*, *G. max*, and *D. lablab* (Leguminosae) in this study were lower compared to *V. faba*. Although in comparison to leaf vegetables growing in the same area [12], Cd content and exceedance of fruit vegetables in this study were lower, the influence of atmospheric deposition cannot be ignored [29].

**Figure 2:**
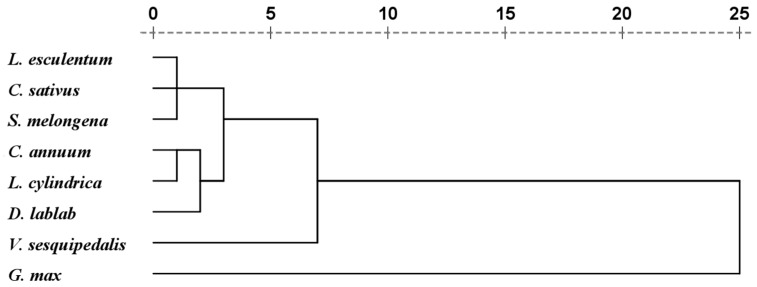
Cluster analysis of the average concentrations of trace elements (based on fresh weight) in different garden vegetables grown in the study area (*L. esculentum*: *Lycopersicon esculentum*; *C. sativus*: *Cucumis sativus*; *S. melongena*: *Solanum melongena*; *C. annuum*: *Capsicum annuum*; *L. cylindrica*: *Luffa cylindrica*; *D. lablab*: *Dolichos lablab*; *V. sesquipedalis*: *Vigna sesquipedalis*; *G. max: Glycine max*).

The information about the relationships among the different vegetables in this study is presented in Figure 2. The cluster diagram showed five main clusters: the first cluster includes *L. esculentum*, *C. sativus*, and *S. melongena*; the second cluster contains *C. annuum* and *L. cylindrica*; the third cluster contains *D. lablab*; the forth cluster contains *V. sesquipedalis*; and the fifth cluster contains *G. max*. These dendrograms explained the grouping of vegetables of similar or nearly identical extraction behavior. The cluster analysis of the average contents of trace elements in different vegetables also showed that the species of Leguminosae had different trace element accumulation properties to other vegetables.

The correlation coefficient among five trace elements in the vegetables is shown in Table S1. In all of the eight vegetables investigated, the correlation coefficient among the five trace elements in the vegetable fruits was not always significantly positive correlated, except that between Cu and Zn. For example, in three vegetables belonging to Solanaceae, although *S. melongena* and *L. esculentum* displayed a significantly positive relationship among the five trace elements, there was not any significantly positive relationship between As and Zn/Pb/Cd for *C. annuum*. The above results indicated that both plant species and chemical elements affected the contents of trace element in vegetable fruits, which was also found by Soudek et al. [30]. In fact, plant absorption and transfer for different trace elements were different [31].

The relationship between the trace element contents in soil and in fruit vegetables is inconsistent for the different vegetables and trace elements. In the eight fruit vegetables investigated, there was a significant relative relationship between the Cd contents in soil and in fruit, except for the vegetables of *G. max*, *D. lablab*, and *C. sativus*. However, the significant relative relationship was only found between the contents in soil and in fruits of *V. sesquipedalis* for Pb. In the three leguminous vegetables, no significant relative relationship was found between the contents of the five trace elements in soils and in fruits for *G. max* and *D. lablab* (Table S2).

### 3.3. Trace Element Transfer from Soil to Vegetables

The transfer factor (*TF*) of trace elements from soil to plants is defined as the ratio of trace element contents in plant (DW) to the total trace element contents in soil (DW) [27]. As seen in Figure S1, large variations in *TF* were observed among different vegetables and trace elements. In the eight fruit vegetables investigated, the mean *TF* values for Cu, Zn, Pb, Cd, and As ranged from 0.10 to 0.27, 0.17 to 0.48, 0.001 to 0.017, 0.02 to 4.3, and 0.002 to 0.042, respectively, which were consistent with or higher than published reports [29]. For the five trace elements, the mean *TF* displayed the following order: Cd > Zn > Cu > As > Pb, which was similar to the results of Xu et al. [12]. In the eight fruit vegetables investigated, the *C. annuum*, *S. melongena*, and *L. esculentum* belonging to Solanaceae had higher *TF* of Cd, which indicated that the solanaceous vegetables were more likely to accumulate Cd in their fruits compared with leguminous and cucurbitaceous vegetables. In the three solanaceous vegetables, the mean *TF* followed the following order: *S. melongena* (2.428) > *C. annuum* (0.576) > *L. esculentum* (0.417). These results were also confirmed by Cd contents in fruits and exceedance of allowable threshold shown in Table 3. The higher *TF* implied higher health risks when these solanaceous vegetables, especially *S. melongena*, were planted in soil contaminated by Cd. Compared to the other trace elements, the average *TF* of Pb was the lowest in this study and less than that reported by Rehman et al. [32]. However, the Pb content in some fruits of *L. esculentum* and *D. lablab* vegetables still exceeded the maximum allowable level (Table 3), although mean Pb content in soil was 85.1 mg kg^−1^ (Table 1), which was less than the environmental quality standard for soil (Grade II) in China. Data analysis found that both *L. esculentum* (0.014) and *D. lablab* (0.017) had higher *TF* values for Pb than for the other vegetables (0.001–0.008). Higher mean *TF* values implied that the Pb in soil was easily transferred to the fruits of *L. esculentum* and *D. lablab*.

Compared to other vegetables in this study, although *S. melongena* had a significantly higher *TF* for Cd, the transfer factor for Zn was significantly lower. This result was similar to the report of Zhuang et al. [33], who suggested that Cd could bind with enzymes instead of Zn, because Zn and Cd could affect nucleic acid metabolism in the same manner. In the eight fruit vegetables investigated, *G. max* had the highest mean *TF* for Cu, Zn, and As and the lowest *TF* of Pb and Cd. This result suggests that *G. max* may selectively transfer trace elements from soil to edible parts of plants.

In this study, the significant difference in *TF* among eight vegetables for the same trace element or five trace elements for the same vegetable may be attributed to many aspects, such as vegetable species and their physiological characters [34], physico-chemical properties of pollutants, and/or nutrient management of soil properties [29].

### 3.4. Health Risk of Inhabitant via Consuming Vegetables

The estimated daily intakes (EDIs) of Cu, Zn, Pb, Cd, and As were evaluated according to the average content of each element in vegetables and the consumption rate for adults and children. The mean EDIs of Cu, Zn, Pb, Cd, and As from all vegetables were 3.030, 10.649, 0.052, 0.049, and 0.066 mg/day for adults and 7.736, 27.190, 0.132, 0.126, and 0.168 mg/day for children. For both adults and children, EDIs gradually changed in the following order: Zn > Cu > As > Pb > Cd. The EDI of Cu, Cd, and As in all the vegetable samples was found to be lower, while Pb was found to be higher than those EDIs of vegetables grown in Bangladesh [17]; the EDIs of Cu, Pb, Cd, and As were 4.88, 0.016, 0.019, and 0.013 μg day^−1^ kg^−1^ in fruit vegetable for adults in Huainan [35]; exposures to As, Cd, and Pb via the intake of vegetables were 0.293, 0.396, and 2.00 μg day^−1^ kg^−1^, respectively, in Zhejiang [36]. The total mean intakes of Cd, Pb, and As were 6.9, 104, and 77.4 ng day^−1^ kg^−1^, respectively, for adults in Shanghai [37]. The mean exposure EDIs for Cd, Pb, and As were reported to be 0.066, 0.233, and 0.076 μg day^−1^ kg^−1^, respectively, in Xiamen [38]. The maximum tolerable daily intake (MTDI) of the studied trace elements from consumption of vegetables are 30, 60, 0.21, 0.021, and 0.13 mg day^−1^ for Cu, Zn, Pb, Cd, and As, respectively [17]. Daily intakes of all the trace elements were less than the MTDI, except the EDI of Cd for adults, and the EDI of Cd and As for children. This result revealed that Cd and As had the highest potential health risks via vegetable consumption in the study area.

The non-carcinogenic and carcinogenic risks from consumption of vegetables by adults and children were assessed based on the target hazard quotient (THQ). The target hazard quotients (THQ) of the five trace elements are listed in Table 4. Table 4 shows that the THQ of all the trace elements were <1 for all vegetable species, except Zn in *S. melongena* (1.181) and *G. max* (1.246) for children. The THQ values for individual elements from individual vegetables were <1, suggesting that people would not experience significant health risks if they only ingested individual trace elements from one type of vegetable [39].

**Figure 3:**
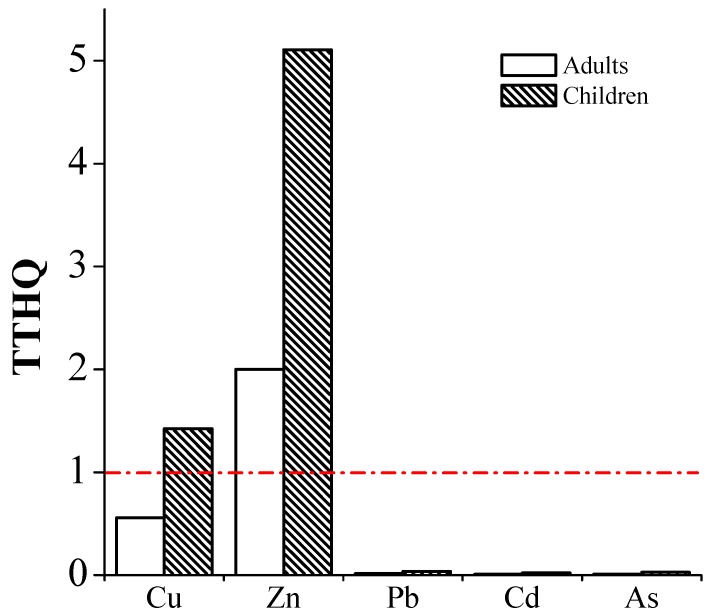
Total target hazard quotient (TTHQ) of trace elements from consuming vegetables of adults and children (Horizontal dotted red line indicates the threshold value of hazard quotient).

The total THQ (TTHQ) values of individual trace elements and individual vegetables were calculated and are given in Table 4. The TTHQ values for the five trace elements through vegetable consumption by the residents in the mining area are presented in Figure 3. The TTHQ value of Zn was the highest for both adults (2.000) and children (5.106), and the value of Cu for children was also higher than 1, suggesting that more attention should be directed toward the ingestion of Zn and Cu. The ranking orders of TTHQ for adults and children were all *G. max* > *S. melongena* > *V. sesquipedalis* > *D. lablab* > *C. annuum* > *L. cylindrica* > *L. esculentum* > *C. sativus*. TTHQ was found to be >1 in *G. max* and *S. melongena* only for children, indicating that children might experience a greater potential health risk from the consumption of these vegetables; therefore, the consumption of these vegetables is considered to be unsafe and their consumption on a regular basis is not recommended.

The HI value expresses the combined noncarcinogenic effects of multiple elements. For the consumption of the selected vegetables, HI was >1 (Table 4) for both adults and children, indicating that consumers may experience adverse health effects from vegetable consumption [17]. By comparing the results with other studies, the health risks for adults and children from consuming vegetables in the study area are comparable to, or even higher than, those associated with the consumption of vegetables [40,41]. The potential non-carcinogenic risks for local residents through vegetable consumption should not be overlooked, although these results are still uncertain because of the nature of the risk assessment [42].

## 4. Conclusions

This study indicated that mining resulted in the pollution of trace elements in the soils. In the study area, Leguminosae vegetables, especially *Glycine max*, had different accumulation characteristics and absorbed Cu, Zn, and As more readily than other vegetables. Cu, Zn, and Cd were highly absorbed by fruit vegetables as compared to other trace elements. As for TTHQ, values of Cu and Zn were >1, indicating that people, especially children, would experience significant health risks if they ingest these two vital elements through the consumption of the studied vegetables. This study calculated the EDI of elements from vegetables by adult and children in the Tongling mining area and the health risk implications from consuming them in terms of THQ and HI. The EDIs of all the elements through the consumption of vegetables were below the MTDI levels, except the EDI of Cd for adults, and the EDI of Cd and As for children. It is therefore suggested that *S. melongena* and *G. max* from the contaminated locations should not be planted and consumed without proper treatment. Cucurbitaceous vegetables grown in this area are relatively safer according to the evaluation of this study. Consequently, some protective measures should be adopted in order to target contamination management, residents’ health protection, crop production safety, and environmental remediation in this area.

## Figures and Tables

**Table 1:** Characteristics of soils collected in the study area.

Soil Type	Parameters	pH	SOM (%) ^a^	Total Content (mg·kg^−1^)				
				Cu	Zn	Pb	Cd	As
Brown red soil	Mean	6.9	2.7	194.7	192.3	85.1	0.48	37.9
(Ferralsols)	Median	7.1	2.3	63.4	139.0	51.6	0.31	17.6
	Range	4.3–9.0	0.7–10.5	18.2–3515.2	64.6–807.6	25.6–441.0	0.05–3.42	1.7–538.5
	CV% ^b^	15.9	51.9	207.6	69.0	95.2	4.9	215.5
GB 15618-1995 ^c^		6.5–7.5		100	250	300	0.6	30

Notes: ^a^ Soil Organic Matter; ^b^ coefficient of variation; ^c^ Grade II of Environmental Quality Standard for soil (GB 15618-1995) in China.

**Table 2:** Correlation coefficient (*r*) of trace elements in soils (*n* = 117).

Elements	Cu	Zn	Pb	Cd	As
Cu	1.000				
Zn	**0.749**	1.000			
Pb	**0.640**	**0.878**	1.000		
Cd	**0.727**	**0.816**	**0.751**	1.000	
As	**0.718**	**0.600**	**0.432**	**0.602**	1.000

Note: Bold *r*-values are significant at *p* < 0.01 level.

**Table 3:** Contents of trace elements in the fruit vegetables. (mg kg^−1^)

Family	Vegetable Species		Cu	Zn	Pb	Cd	As
**Solanaceae**	*C. annuum* (*n* = 94)	Mean (CV%) ^a^	1.083 (32.2) BC ^b^	2.706 (38.7) D	0.010 (80.0) C	0.020 (106.9) AB	0.043 (67.4) B
		Range	0.448–2.756	1.301–7.769	0.001–0.041	0.004–0.155	0.003–0.116
		EAT ^c^/percent	0	0	0	5	0
	*S. melongena* (*n* = 80)	Mean (CV%)	0.861 (91.4) C	1.835 (95.6) E	0.008 (187.5) C	0.051 (374.5) A	0.017 (123.5) C
		Range	0.192–7.281	0.371–15.899	0.001–0.089	0.003–1.707	0.001–0.161
		EAT/percent	0	0	0	14	0
	*L. esculentum* (*n* = 30)	Mean (CV%)	0.528 (36.9) D	1.631 (34.8) E	0.047 (259.6) B	0.016 (75.0) AB	0.004 (100.0) C
		Range	0.226–1.177	0.889–3.781	0.002–0.546	0.003–0.061	0.001–0.016
		EAT/percent	0	0	10	3	0
**Leguminosae**	*V. sesquipedalis* (*n* = 80)	Mean (CV%)	1.222 (29.1) B	5.650 (24.2) B	0.018 (50.0) C	0.006 (116.7) B	0.010 (80.0) C
		Range	0.577–2.720	3.413–10.144	0.001–0.044	0.001–0.055	0.002–0.050
		EAT/percent	0	0	0	0	0
	*G. max* (*n* = 38)	Mean (CV%)	3.614 (38.6) A	15.387 (29.3) A	0.015 (86.7) C	0.022 (54.5) AB	0.106 (68.9) A
		Range	1.095–7.399	4.758–24.914	0.001–0.060	0.007–0.061	0.001–0.243
		EAT/percent	0	0	0	0	0
	*D. lablab* (*n* = 30)	Mean (CV%)	1.062 (35.5) BC	3.607 (32.3) C	0.098 (126.5) A	0.010 (60.0) B	0.009 (55.6) C
		Range	0.071–2.138	0.322–6.360	0.010–0.496	0.000–0.026	0.000–0.024
		EAT/percent	0	0	17	0	0
**Cucurbitaceae**	*L. cylindrical* (*n* = 65)	Mean (CV%)	0.846 (31.7) C	2.852 (33.5) D	0.007 (57.1) C	0.004 (75.0) B	0.005 (120.0) C
		Range	0.003–1.461	0.007–4.805	0.001–0.015	0.000–0.016	0.000–0.046
		EAT/percent	0	0	0	0	0
	*C. sativus* (*n* = 24)	Mean (CV%)	0.361 (46.8) D	1.539 (45.0) E	0.018 (133.3) C	0.004 (75.0) B	0.013 (69.2) C
		Range	0.159–0.999	0.487–4.375	0.005–0.092	0.001–0.018	0.004–0.032
		EAT/percent	0	0	0	0	0
	Maximum allowable levels in food ^d^		-	-	0.1 ^e^ 0.2 ^f^	0.05 ^e^ 0.1 ^f^	0.5

Notes: ^a^ CV%: coefficient of variation; ^b^ A, B, C, D, E: Means within the same column with the same capital letter are not significantly different (*p* < 0.05); ^c^ EAT: Exceedance of allowable threshold; ^d^ Tolerance limit of contaminants in food (GB 2762-2012); ^e^ Maximum allowable levels in fresh vegetables except leguminous vegetables; ^f^ Maximum allowable levels in leguminous vegetables.

**Table 4:** Target hazard quotient (THQ; noncarcinogenic risk) of trace elements from consuming vegetables.

Vegetables	THQ											
	Cu		Zn		Pb		Cd		As		TTHQ	
	Adult	Children	Adult	Children	Adult	Children	Adult	Children	Adult	Children	Adult	Children
*C. annuum*	0.070	0.179	0.175	0.446	6.169 × 10^−4^	1.575 × 10^−3^	1.288 × 10^−3^	3.288 × 10^−3^	2.762 × 10^−3^	7.052 × 10^−3^	2.497 × 10^−1^	6.369 × 10^−1^
*S. melongena*	0.123	0.313	0.463	1.181	5.520 × 10^−4^	1.409 × 10^−3^	3.086 × 10^−3^	7.879 × 10^−3^	3.482 × 10^−3^	8.890 × 10^−3^	5.931 × 10^−1^	1.512
*L. esculentum*	0.034	0.087	0.105	0.269	3.017 × 10^−3^	7.702 × 10^−3^	1.014 × 10^−3^	2.590 × 10^−3^	2.459 × 10^−4^	6.279 × 10^−4^	1.433 × 10^−1^	3.669 × 10^−1^
*V. sesquipedalis*	0.068	0.173	0.292	0.746	8.839 × 10^−4^	2.257 × 10^−3^	2.844 × 10^−4^	7.262 × 10^−4^	4.973 × 10^−4^	1.270 × 10^−3^	3.617 × 10^−1^	9.233 × 10^−1^
*G. max*	0.116	0.297	0.488	1.246	1.255 × 10^−3^	3.205 × 10^−3^	6.896 × 10^−4^	1.761 × 10^−3^	2.106 × 10^−3^	5.378 × 10^−3^	6.081 × 10^−1^	1.553
*D. lablab*	0.069	0.175	0.233	0.595	6.309 × 10^−3^	1.611 × 10^−2^	6.252 × 10^−4^	1.596 × 10^−3^	5.614 × 10^−4^	1.433 × 10^−3^	3.095 × 10^−1^	7.891 × 10^−1^
*L. cylindrica*	0.054	0.138	0.145	0.369	6.085 × 10^−4^	1.554 × 10^−3^	1.095 × 10^−3^	2.797 × 10^−3^	3.431 × 10^−4^	8.760 × 10^−4^	2.010 × 10^−1^	5.122 × 10^−1^
*C. sativus*	0.023	0.060	0.099	0.254	1.144 × 10^−3^	2.920 × 10^−3^	2.605 × 10^−4^	6.652 × 10^−4^	8.663 × 10^−4^	2.212 × 10^−3^	1.243 × 10^−1^	3.198 × 10^−1^
TTHQ ^a^	0.557	1.422	2.000	5.106	1.439 × 10^−2^	3.673 × 10^−2^	8.343 × 10^−3^	2.130 × 10^−2^	1.086 × 10^−2^	2.774 × 10^−2^	HI ^b^ = 2.591	HI = 6.614

Notes: ^a^ TTHQ (Total THQ); ^b^ HI (Hazard Index).

## Supplementary Materials

The following are available online at http://www.mdpi.com/1660-4601/15/2/202/s1, Figure S1: Box-Whisker plots of the transfer factor (TF) of heavy metals from soil to the edible part of different vegetables, Table S1: Correlation coefficient (*r*) of trace elements in the vegetables, Table S2: Correlation coefficients between the trace element contents in fruit vegetables (mg kg^−1^, based on fresh weight) and in soil.
