# Supplementary material for: Trace Elements in Soils and Selected Agricultural Plants in the Tongling Mining Area of China
Source: Int J Environ Res Public Health. 2018 Jan 25;15(2):202. doi: 10.3390/ijerph15020202 (PMC5858271; doi:10.3390/ijerph15020202)
Supplement: Supplementary file 1 [file ijerph-15-00202-s001.zip › Table S2.pdf]

Table S2

Correlation coefficients between the trace element contents in fruit vegetables(mg kg^{-1},based on fresh weight) and in soil.

	Cu	Zn	Cd	Pb	As
C. annuum	0.18	0.114	0.318**[a]	0.123	-0.023
V. sesquipedalis	0.132	0.309**	0.403**	0.236*	0.360**
S. melongena	0.042	0.197	0.463**	0.143	0.158
L. cylindrica	0.045	0.144	0.439**	0.017	0.085
G. max	-0.053	0.307	0.230	0.078	0.095
D. lablab	-0.121	0.241	0.247	0.305	0.191
L. esculentum	0.327	0.106	0.663**	-0.154	0.205
C. sativus	0.070	-0.048	0.050	-0.096	0.582**

Note: [a] * and **, Correlation is significant at the 0.05 and 0.01 probability levels, respectively.
