# Supplementary material for: Trace Elements in Soils and Selected Agricultural Plants in the Tongling Mining Area of China
Source: Int J Environ Res Public Health. 2018 Jan 25;15(2):202. doi: 10.3390/ijerph15020202 (PMC5858271; doi:10.3390/ijerph15020202)
Supplement: Supplementary file 1 [file ijerph-15-00202-s001.zip › Figure S1.pdf]

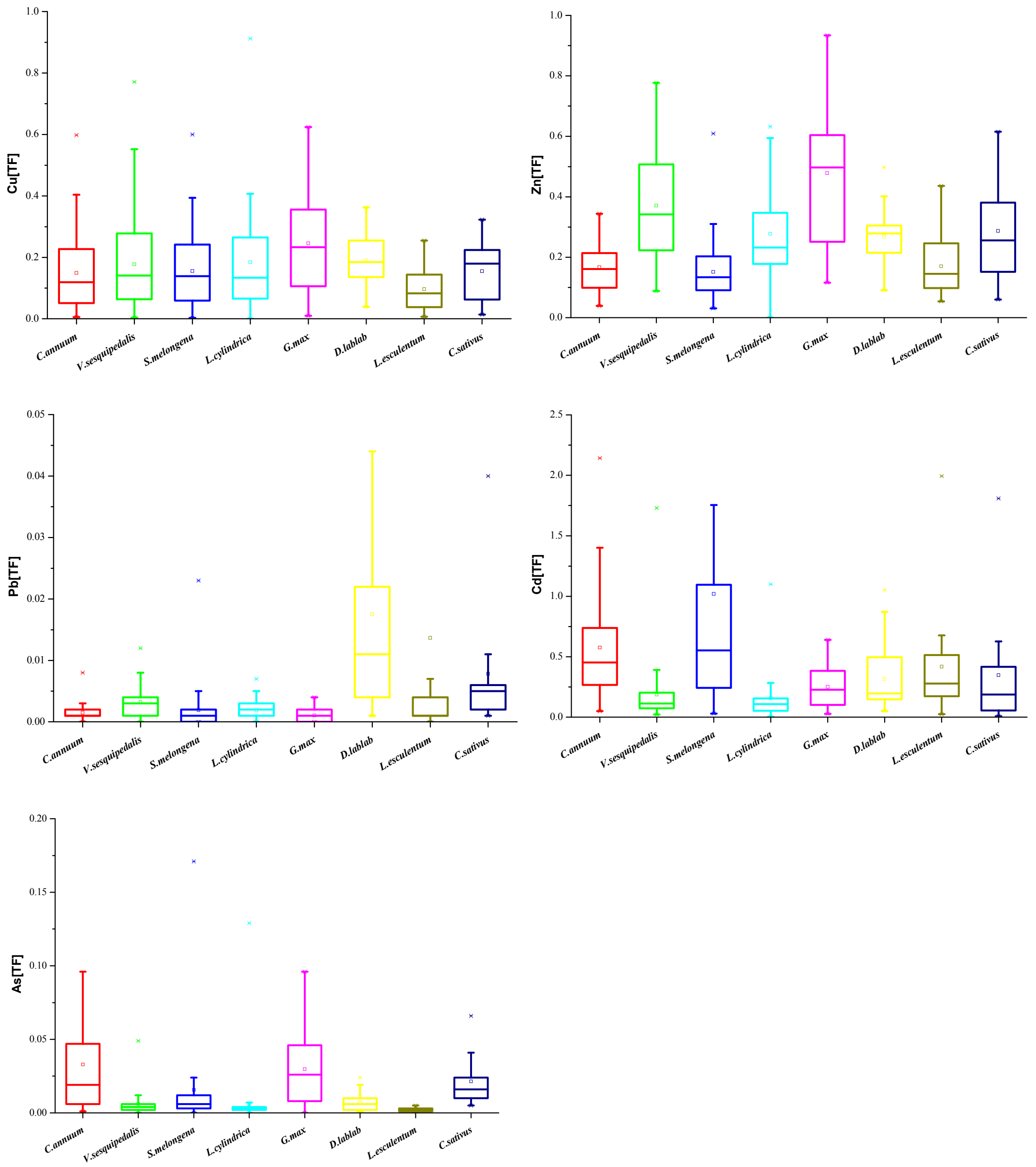

Figure S1. Box-Whisker plots of the transfer factor (TF) of heavy metals from soil to the edible part of different vegetables.
