# Supplementary material for: Trace Elements in Soils and Selected Agricultural Plants in the Tongling Mining Area of China
Source: Int J Environ Res Public Health. 2018 Jan 25;15(2):202. doi: 10.3390/ijerph15020202 (PMC5858271; doi:10.3390/ijerph15020202)
Supplement: Supplementary file 1 [file ijerph-15-00202-s001.zip › Table S1.pdf]

Table S1 Correlation coefficient (*r*) of trace elements in the vegetables.

	Cu	Zn	Pb	Cd	As
Cu	1.000				
Zn	**0.910**[a]	1.000			
Pb	**0.642**	**0.628**	1.000		
Cd	**0.329**	**0.151**	**0.212**	1.000	
As	0.044	0.057	-0.024	0.070	1.000

Note: [a] Bold *r*-values are significant at $p < 0.01$.
